# Oral anticoagulation versus antiplatelet therapy for secondary stroke prevention in patients with embolic stroke of undetermined source: A systematic review and meta-analysis

**DOI:** 10.1177/23969873221076971

**Published:** 2022-02-11

**Authors:** Nikhil Nair Hariharan, Kashyap Patel, Omaike Sikder, Kanjana S Perera, Hans-Christoph Diener, Robert G Hart, John W Eikelboom

**Affiliations:** 1 Michael G. DeGroote School of Medicine, 3710 McMaster University, Hamilton, ON, Canada; 2 School of Medicine, University of Ottawa, Ottawa, ON, Canada; 3 3710 McMaster University, Hamilton, ON, Canada; 4 Hamilton Health Sciences, 3710 McMaster University, Hamilton, ON, Canada; 5 33493 Population Health Research Institute, Hamilton, ON, Canada; 6 123109 University of Duisburg-Essen, Duisburg, Germany

**Keywords:** Anticoagulation, antiplatelet, stroke, prevention, embolic stroke of undetermined source

## Abstract

**Purpose:**

We performed a systematic review and meta-analysis of randomized controlled trials (RCTs) to evaluate the efficacy and safety of direct oral anticoagulation (DOAC) compared with antiplatelet therapy for secondary stroke prevention in adult patients with embolic stroke of undetermined source (ESUS).

**Method:**

We searched major databases (Embase, MEDLINE, CINAHL, CENTRAL, and Web of Science) for RCTs published until March 2021. The primary outcome was recurrent stroke, and the main safety outcomes were major bleeding and clinically relevant non-major bleeding (CRNB). We assessed risk of bias using the Cochrane Risk of Bias tool. We used a random-effects model to determine pooled risk ratios and 95% confidence intervals in the datasets and key subgroups.

**Findings:**

Our search identified two RCTs, involving a total of 12,603 patients with ESUS. Anticoagulation with dabigatran or rivaroxaban compared with aspirin did not reduce the risk of recurrent stroke (RR, 0.96 [0.76–1.20]) or increase major bleeding (RR, 1.77 [0.80–3.89]) but significantly increased the composite of major or clinically relevant non-major bleeding (RR, 1.57 [1.26–1.97]). Prespecified subgroup analysis demonstrated consistent results according to age and sex. Additional post-hoc subgroup analyses demonstrated consistent results according to prior stroke and presence of a patent foramen ovale but suggested that DOACs reduced recurrent stroke in patients with an estimated glomerular filtration rate (eGFR) <50 and 50-80 ml/min but not in those with eGFR >80 ml/min (interaction *P* = 0.0234).

**Discussion/conclusion:**

Direct oral anticoagulations are not more effective than aspirin in preventing stroke recurrence in patients with ESUS and increase bleeding.

**Registration:**

PROSPERO ID: CRD42019138593

## Introduction

Ischemic strokes account for about 80% of all strokes.^1^ Most ischemic strokes are caused by atherosclerosis or embolism from the heart, but approximately one-third are of uncertain cause and are often referred to as cryptogenic.^2,3^ Embolic stroke of undetermined source (ESUS) is a subset of cryptogenic strokes characterized by non-lacunar ischemic infarction without an identifiable proximal artery or cardiac source of embolism.^4^ Randomized controlled trials (RCTs) in patients with ESUS did not show benefit of direct oral anticoagulation (DOAC) compared with antiplatelet therapy for prevention of recurrent stroke, but secondary analyses have raised the possibility that there might be a benefit in certain subgroups, including older patients and those with a patent foramen ovale.^5,6^ We performed a systematic review and meta-analysis of randomized controlled trials to obtain best estimates of the efficacy and safety of oral anticoagulation compared with antiplatelet therapy for secondary stroke prevention in adult patients with ESUS and in key patient subgroups.

## Materials and methods

This systematic review and meta-analysis were performed according to the Preferred Reporting Items for Systematic Reviews and Meta-Analyses (PRISMA) guidelines.^7^ The protocol was registered in the PROSPERO international prospective register of systematic reviews (CRD42019138593).

### Eligibility criteria

We included randomized controlled trials that compared the efficacy and safety of anticoagulant and antiplatelet therapy for secondary stroke prevention in adult patients with ESUS. We did not include any time or language restrictions. Sensitive eligibility criteria were used for the title and abstract screening. All studies that report stroke recurrence and major bleeding were selected for full-text screening. A study was included in this meta-analysis if it fulfilled 3 predefined criteria: (1) designed as a randomized controlled trial comparing anticoagulant to antiplatelet therapy in ESUS patients; (2) reported quantitative data on recurrent stroke, major bleeding, and clinically relevant non-major bleeding; and (3) was published up to March 1st, 2021.

### Search strategy

The search strategy was developed with the assistance of a clinical health sciences librarian experienced in reviews. Systematic searches were conducted in Embase, MEDLINE, CINAHL, Cochrane Central, and Web of Science from inception to March 1st, 2021. The last search was performed on March 1st, 2021. The full electronic search strategy used for every database is available in the supplement.

### Study selection

Two reviewers (N.N.H. and O.S.) independently assessed all titles and abstracts for relevance according to the sensitive eligibility criteria. When duplicates were identified, the most recent study was included. Full-text screening was performed by the same two reviewers (N.N.H. and O.S.) in accordance with stricter eligibility criteria. Reviewers (N.N.H. and O.S.) also manually reviewed the reference lists of the included studies to identify further potentially eligible articles. Disagreements during screening were resolved by a third reviewer (K.P.).

### Data extraction

Two reviewers (N.N.H. and O.S.) independently extracted the relevant data from the eligible studies into a standardized Microsoft Excel file. Disagreements were resolved following discussion, and the final decision was reached via consensus with the third reviewer (K.P.). The extracted data included study design and characteristics (first author, date of publication, and country of origin); number, sex, age, and comorbidities of patients.

### Outcomes

The primary outcome was recurrent stroke. Secondary outcomes included ischemic stroke, disabling stroke, systemic embolism, myocardial infarction, and all-cause mortality. The safety outcomes were major bleeding, clinically relevant non-major bleeding, and hemorrhagic stroke.

### Risk of bias assessment

Risk of bias was assessed independently by two reviewers (O.S. and K.P.) using the Cochrane Risk of Bias Tool.^8^ The following quality criteria were evaluated for having low, high, or unclear risk of bias: sequence generation, allocation concealment, blinding, incomplete outcome data, and selective outcome reporting. Discrepancies were resolved via reviewing studies in consensus.

### Data synthesis

Review Manager 5.4 software by Cochrane Reviews was used to perform the statistical analysis for this meta-analysis.^9^ Risk ratios (RRs) with 95% confidence intervals were calculated for the dichotomous outcomes. A random-effects model was used for analysis. I^2^ values were calculated for each reported outcome to determine heterogeneity. If the I^2^ < 25%, that outcome was considered to have low heterogeneity, 25% < I^2^ < 75% was considered as moderate heterogeneity, and I^2^ > 75% was considered as high/significant heterogeneity. Prespecified subgroup analyses included age and sex, and additional post-hoc subgroup analyses were performed for renal function, history of stroke, and patent foramen ovale (PFO).

## Results

**Figure 1.**
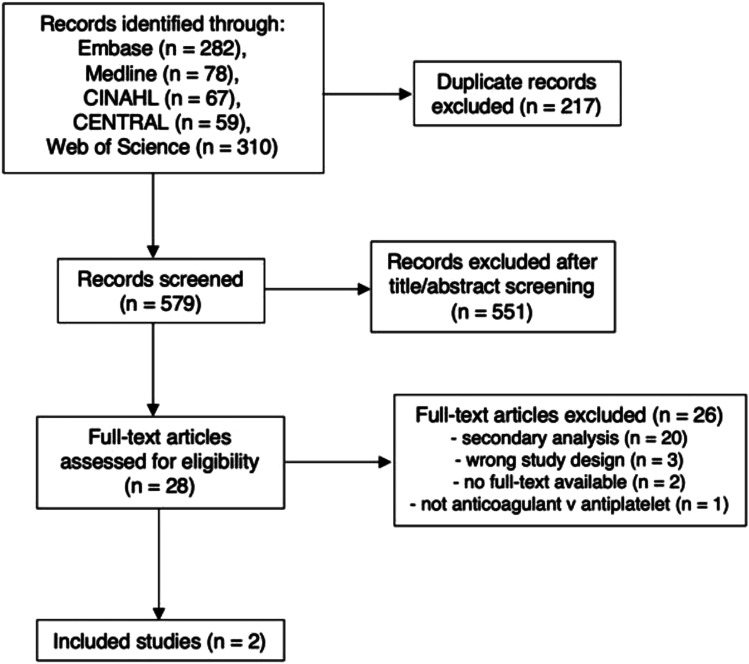
PRISMA flow diagram.

The literature search yielded 579 unique records. After screening titles and abstracts, 28 articles were retrieved for full-text evaluation; 2 studies satisfied the predetermined eligibility criteria and were included in this meta-analysis as shown in the PRISMA flow diagram (Figure 1).

### Study characteristics

The studies included a combined total of 12, 603 patients with a mean age of 65.6 years and 62.5% males. 24% of patients had diabetes mellitus, 75.9% had hypertension, 18.8% of patients were actively using tobacco, and 17.75% of patients had a previous history of stroke or TIA. Additional details of eligibility criteria and baseline characteristics are presented in Supplemental Tables I–III.

### Quality assessment

Based on the Cochrane Risk of Bias Tool, the methodological quality of both studies was high. Details of the quality assessments can be found in Supplemental Table IV.

### Follow-up and outcomes

Details of the outcomes in each trial can be found in Supplemental Table V. Patients were followed for a median of 11 months in NAVIGATE-ESUS (rivaroxaban versus aspirin) and 19 months in RE-SPECT ESUS (dabigatran versus aspirin).

### Recurrent stroke

**Figure 2.**
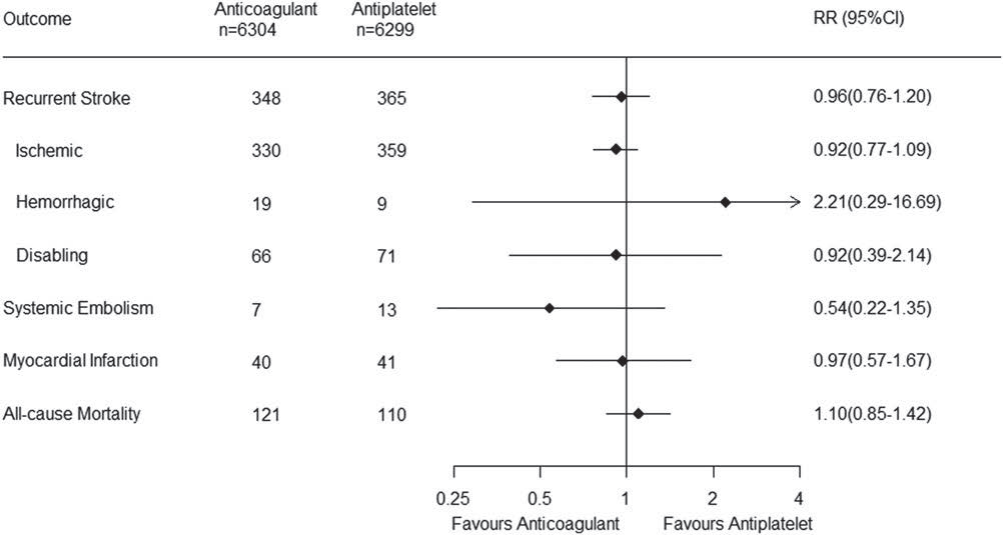
Forest plot of primary and secondary outcomes. RR, risk ratio.

Recurrent stroke occurred in 713 patients (5.7%): 348 (5.5%) in the anticoagulant group treated and 365 (5.8%) in the antiplatelet group. Anticoagulant therapy did not reduce the risk of recurrent stroke (RR, 0.96 [95% CI 0.76–1.20]; *P* = 0.71; I^2^ = 61%) (Figure 2).

### Secondary outcomes

Anticoagulants compared to antiplatelet therapy did not reduce the risk of ischemic stroke (RR, 0.92 [0.77–1.09]; *P* = 0.35; I^2^ = 29%), disabling stroke (RR, 0.92 [0.39–2.14]; *P* = 0.85; I^2^ = 84%), systemic embolism (RR, 0.54 [0.22–1.35]; *P* = 0.19; I^2^ = 0%), myocardial infarction (RR, 0.97 [0.57–1.67]; *P* = 0.92; I^2^ = 34%), or mortality (RR, 1.10 [0.85–1.42]; *P* = 0.47; I^2^ = 0%) (Figure 2). There was also no reduction in the composite of recurrent stroke or systemic embolism (RR, 0.95 [0.76–1.19]; *P* = 0.68; I^2^ = 60%).

### Safety outcomes

**Figure 3.**
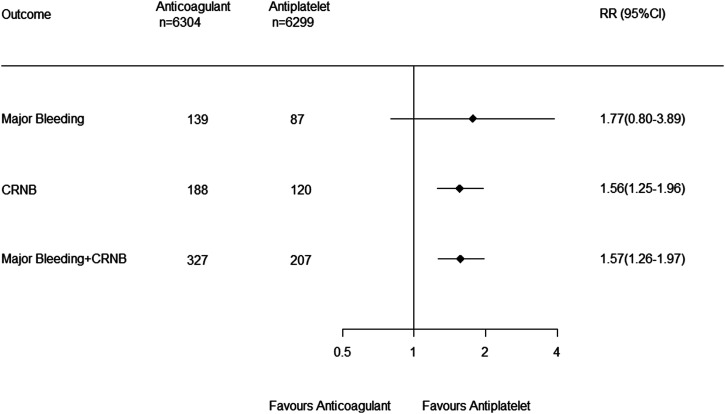
Forest plot of safety outcomes. RR, risk ratio; CRNB, clinically relevant non-major bleeding.

Major bleeding occurred in 226 patients (1.8%): 139 (2.2%) in the anticoagulant group and 87 (1.4%) in the antiplatelet group. Anticoagulation compared to antiplatelet therapy did not increase the risk of major bleeding (RR, 1.77 [0.80–3.89]; *P* = 0.16; I^2^ = 28%) (Figure 3). CRNB occurred in 308 patients (2.4%): 188 (3.0%) in the anticoagulant group and 120 (1.9%) in the antiplatelet group. Anticoagulant compared to antiplatelet therapy significantly increased CRNB (RR, 1.56 [1.25–1.96]; *P* = 0.0001; I^2^ = 31%) as well as the composite of major or CRNB (RR, 1.57 [1.26–1.97]; *P* < 0.0001; I^2^ = 43%) (Figure 3) but did not increase hemorrhagic stroke (RR, 2.21 [0.29–16.69]; *P* = 0.44; I^2^ = 79%) (Figure 2).

### Subgroup analyses

**Figure 4.**
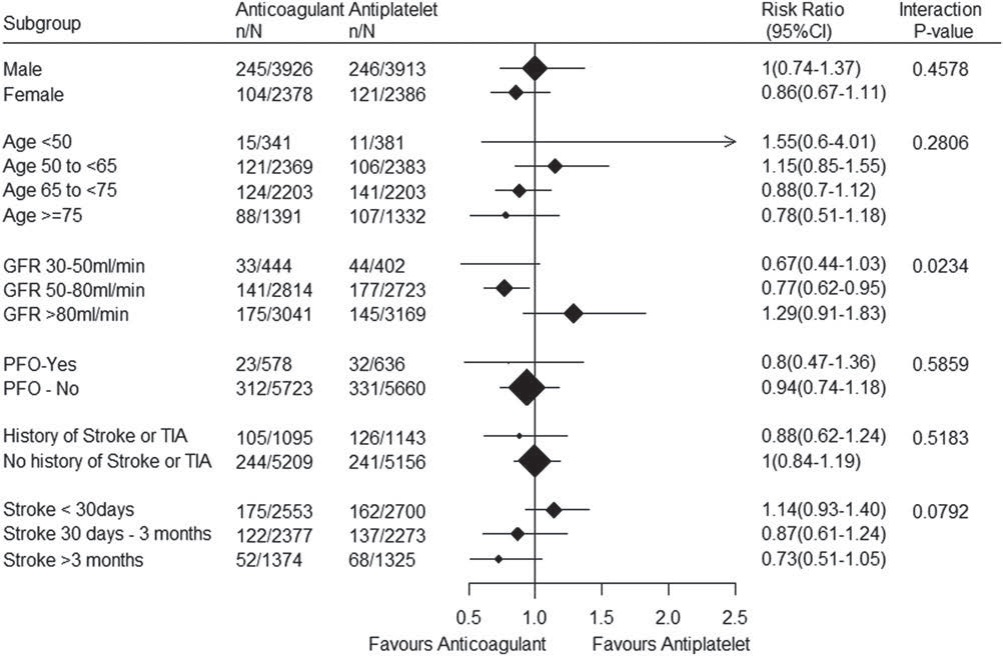
Forest plot of subgroup analyses for recurrent stroke. RR, risk ratio; GFR, glomerular filtration rate; PFO, patent foramen ovale; TIA, transient ischemic stroke.

Anticoagulants compared with antiplatelet therapy produced similar effects on recurrent stroke in prespecified subgroups defined by age and sex. Effects were also consistent in post-hoc subgroups defined by the presence or absence of PFO and history of stroke or TIA, but anticoagulants appeared to reduce recurrent stroke in patients with mild or moderate chronic kidney disease and not those with preserved renal function (interaction *P* = 0.0234) (Figure 4). There was also a suggestion of benefit of anticoagulation compared with aspirin in patients randomized beyond 30 days after stroke compared with those randomized within the first 30 days, although the test for interaction was not statistically significant. The effects of anticoagulation compared with antiplatelet therapy on major bleeding were consistent in patients with or without PFO (interaction P=0.5736). Bleeding data were not available for other subgroups.

## Discussion

The results of our meta-analysis provide no evidence that anticoagulation with a DOAC is superior to aspirin for prevention of recurrent stroke in patients with ESUS. There was also no reduction in any of the secondary efficacy outcomes, including ischemic stroke, myocardial infarction, and all-cause mortality. Anticoagulants compared with aspirin significantly increased major or clinically relevant non-major bleeding.

The results of our exploratory subgroup analyses suggest an interaction between randomized treatment and renal function, with no benefit of anticoagulation in ESUS patients with eGFR >80 ml/min (RR 1.29) and progressively lower relative risks in those with eGFR 50–80 ml/min (RR 0.77) or eGFR <50 ml/min (RR 0.67). Patients with renal impairment are generally older than those without renal impairment, and although we did not see a significant interaction between treatment and age, the pattern was consistent with that seen for renal function. In the context of no overall benefit of anticoagulation, the interaction between treatment and renal function must be cautiously interpreted. Several explanations should be considered. First, it is possible that patients with impaired renal function were at higher risk of cardioembolic stroke because of a higher prevalence of subclinical atrial fibrillation, and therefore benefitted from anticoagulation.^10,11^ Second, patients with worse renal function are likely to have had higher blood concentrations of the anticoagulant than those with preserved renal function because both dabigatran and rivaroxaban are least partially renally cleared.^12,13^ It is not known whether higher drug concentrations might be beneficial in patients with ESUS. Third, patients with impaired renal function have reduced pharmacodynamic response to aspirin, possibly related to impaired enteral absorption, chronic inflammation, or concomitant use of nonsteroidal anti-inflammatory drugs.^14^ These explanations remain unproven.

Subgroup analyses also suggest the possibility of an interaction between treatment and the timing of randomization since stroke. There is some evidence that aspirin has a larger benefit during the first 30 days after stroke (about a 60% risk reduction) than during longterm.^15^ It is possible that this contributed to the finding of a trend for increased benefit over time”.

Emerging evidence suggests that PFO closure reduces the risk of recurrent stroke in patients with previous cryptogenic stroke, presumably by preventing paradoxical embolism.^16^ Consistent with this conclusion, subgroup analysis from the NAVIGATE-ESUS trial suggested a benefit of anticoagulation in ESUS patients with PFO. However, no benefit of anticoagulation was seen in the RE-SPECT ESUS trial, and the pooled data from the two trials do not suggest a benefit of anticoagulation in ESUS patients with PFO.^17,18^

The lack of overall benefit of anticoagulation compared with antiplatelet therapy in patients with ESUS highlights the need for high-quality evidence to inform clinical practice. Prior to the results of the randomized trials, clinicians often considered empiric use of anticoagulation in patients with embolic pattern stroke.^19^ Following the results of the NAVIGATE-ESUS and RE-SPECT ESUS trials, updated guidelines now recommend that most patients with ESUS receive antiplatelet therapy rather than anticoagulation.^20^ Despite the routine use of aspirin, however, the risk of recurrent stroke in ESUS patients remains high, highlighting the need for alternative approaches.^21^ ATTICUS is an ongoing randomized trial comparing apixaban with aspirin in patients with ESUS and results are expected in 2022.^22^

### Limitations

This study has several limitations. First, we found only two randomized trials of anticoagulation versus antiplatelet therapy in ESUS. Although our overall findings are therefore not surprising, the results for secondary outcomes and in key subgroups provide new insights. Second, several of the subgroups that are presented were identified post-hoc and determined by the availability of published data. Accordingly, the results of subgroup analyses should be considered hypothesis-generating. Third, we did not have access to bleeding data in all the subgroups or to individual patient data which would have provided greater power to explore subgroups. Fourth, it is likely that many PFOs were undetected because sensitive diagnostic testing using transesophageal echocardiogram and bubble/contrast tests was not routinely performed. Fifth, the median duration of follow-up in the trials was only 11 to 19 months, and it is possible that longer follow-up may have demonstrated significant benefits of treatment, as also suggested by the late divergence of the Kaplan–Meier curves in RE-SPECT ESUS. Finally, the results of our meta-analysis might not be generalizable to other anticoagulant or antiplatelet treatment regimens.

## Supplemental Material

sj-pdf-1-eso-10.1177_23969873221076971 – Supplemental Material for Oral anticoagulation versus antiplatelet therapy for secondary stroke prevention in patients with embolic stroke of undetermined source: A systematic review and meta-analysis Click here for additional data file. Supplemental Material, sj-pdf-1-eso-10.1177_23969873221076971 for Oral anticoagulation versus antiplatelet therapy for secondary stroke prevention in patients with embolic stroke of undetermined source: A systematic review and meta-analysis by Nikhil Nair Hariharan, Kashyap Patel, Omaike Sikder, Kanjana S Perera, Hans-Christoph Diener, Robert G Hart and John W Eikelboom in European Stroke Journal
