# Supplementary material for: Oral anticoagulation versus antiplatelet therapy for secondary stroke prevention in patients with embolic stroke of undetermined source: A systematic review and meta-analysis
Source: Eur Stroke J. 2022 Feb 11;7(2):92–8. doi: 10.1177/23969873221076971 (PMC9134773; doi:10.1177/23969873221076971)
Supplement: sj-pdf-1-eso-10.1177_23969873221076971 – Supplemental Material for Oral anticoagulation versus antiplatelet therapy for secondary stroke prevention in patients with embolic stroke of undetermined source: A systematic review and meta-analysis [file sj-pdf-1-eso-10.1177_23969873221076971.pdf]

SUPPLEMENTAL MATERIAL

Anticoagulant versus antiplatelet therapy for secondary stroke prevention in patients with embolic stroke of undetermined source (ESUS): a systematic review and meta-analysis

Authors: Nikhil Nair Hariharan, BSc1; Kashyap Patel, BHSc2; Omaike Sikder, BSc1; Hans-Christoph Diener, MD3; Sashi Perera, MD4,5; Robert Hart, MD5; John Eikelboom, MBBS5

1 McMaster University, Hamilton, Ontario, Canada

2 Faculty of Medicine, University of Ottawa, Ottawa, Ontario, Canada

3 University of Duisburg-Essen, Duisburg, Germany

4 Hamilton Health Sciences, Hamilton, Ontario, Canada

5 Population Health Research Institute, Hamilton, Ontario, Canada

Correspondence: John W Eikelboom, MBBS, Population Health Research Institute, 237 Barton Street East, Hamilton, Ontario, L8L 2X2, Canada E-mail Address: john.eikelboom@phri.ca

Number of tables and figures: Figures 4

Word count: 2905

Supplemental Methods

CENTRAL Search:

ID Search

1 MeSH descriptor: [Anticoagulants] explode all trees

2 MeSH descriptor: [Fibrinolytic Agents] explode all trees

3 (anticoagulant) (Word variations have been searched) in Trials

4 (antithrombin) in Trials (Word variations have been searched)

5 MeSH descriptor: [Platelet Aggregation Inhibitors] explode all trees

6 MeSH descriptor: [Aspirin] explode all trees

7 (antiplatelet) (Word variations have been searched) in Trials

8 MeSH descriptor: [Stroke] explode all trees

9 (Stroke prevention) (Word variations have been searched) in Trials

10 (stroke recurrence) in Trials (Word variations have been searched)

11 MeSH descriptor: [Ischemic Attack, Transient] explode all trees

12 (Transient ischaemic attack) (Word variations have been searched) in Trials

13 (Cryptogenic stroke) in Trials (Word variations have been searched)

14 (Embolic Stroke) in Trials (Word variations have been searched)

15 ("Embolic stroke of unknown source" or "Embolic stroke of undetermined source" or "ESUS") in Trials (Word variations have been searched)

16 ("randomized controlled trial" or "randomized clinical trial" or "controlled clinical trial" or random* or trial:ti,ab,kw) (Word variations have been searched) in Trials

17 #1 or #2 or #3 or #4

18 #5 or #6 or #7

19 #8 or #9 or #10 or #11 or #12

20 #13 or #14 or #15

21 #17 and #18 and #19 and #20

22 #21 and #16

Web of Science Search:

ID Search

1 (ALL=“anticoagula*”) AND LANGUAGE: (English) AND DOCUMENT TYPES: (Article)

2 (TS=(anticoagulation OR anticoagulant) AND LANGUAGE: (English) AND DOCUMENT TYPES: (Article)

3 (ALL=(antithrombi*)) AND LANGUAGE: (English) AND DOCUMENT TYPES: (Article)

4 (ALL=(antiplatelet*)) AND LANGUAGE: (English) AND DOCUMENT TYPES: (Article)

5 (ALL=(platelet aggregation inhibit*)) AND LANGUAGE: (English) AND DOCUMENT TYPES: (Article)

6 (ALL=(aspirin OR acetylsalicylic acid)) AND LANGUAGE: (English) AND DOCUMENT TYPES: (Article)

7 (ALL=(cryptogenic stroke)) AND LANGUAGE: (English) AND DOCUMENT TYPES: (Article)

8 (ALL=(Embolic stroke of undetermined source OR Embolic stroke of unknown source OR ESUS)) AND LANGUAGE: (English) AND DOCUMENT TYPES: (Article)

9 (TS=(embolic stroke)) AND LANGUAGE: (English) AND DOCUMENT TYPES: (Article)

10 (ALL=(embolic stroke)) AND LANGUAGE: (English) AND DOCUMENT TYPES: (Article)

11 (ALL=(stroke OR stroke prevent*)) AND LANGUAGE: (English) AND DOCUMENT TYPES: (Article)

12 (ALL=(stroke recurrence)) AND LANGUAGE: (English) AND DOCUMENT TYPES: (Article)

13 (ALL=(transient ischemic attack OR transient ischaemic)) AND LANGUAGE: (English) AND DOCUMENT TYPES: (Article)

14 3 OR 2 OR 1

15 6 OR 5 OR 4

16 10 OR 9 OR 8 OR 7

17 13 OR 12 OR 11

18 (17 AND 16 AND 15 AND 15) AND LANGUAGE: (English)

CINAHL Search:

1 (MH “Anticoagulants+”)

2 anticoagula*

3 antithrombin*

4 (MH “Platelet Aggregation Inhibitors+)

5 aspirin

6 acetylsalicylic acid

7 antiplatelet*

8 anti-platelet*

9 1 OR 2 OR 3

10 4 OR 5 OR 6 OR 7 OR 8

11 (MH “Stroke+”)

12 stroke prevention

13 (MH “Cerebral Ischemia+”)

14 stroke recurrence

15 11 OR 12 OR 13 OR 14

16 cryptogenic stroke

17 embolic stroke of undetermined source

18 embolic stroke of unknown source

19 ESUS

20 16 OR 17 OR 18 OR 19

21 9 AND 10 AND 15 AND 20

Embase Search

1 anticoagula*.mp.

2 exp Anticoagulants/

3 anti?platelet.mp.

4 exp Platelet Aggregation Inhibitors/

5 aspirin.mp.

6 acetylsalicylic acid.mp.

7 exp Stroke/

8 stroke prevention.mp.

9 stroke recurrence.mp.

10 exp Ischemic Attack, Transient/

11 cryptogenic stroke.mp.

12 embolic stroke of undetermined source.mp.

13 embolic stroke of unknown source.mp.

14 ESUS.mp.

15 antithrombin.mp. or exp *Antithrombins/

16 1 or 2 or 15

17 3 or 4 or 5 or 6

18 7 or 8 or 9 or 10

19 11 or 12 or 13 or 14

20 16 and 17 and 18 and 19

21 randomized controlled trial.pt.

22 controlled clinical trial.pt.

23 randomized.ab.

24 placebo.ab.

25 clinical trials as topic.sh.

26 randomly.ab.

27 trial.ti.

28 pragmatic clinical trial.pt.

29 “randomized controlled trial (topic)”/

30 randomized controlled trial/

31 randomization/

32 random allocation/

33 double-blind method/

34 double blind procedure/

35 double-blind studies/

36 single-blind procedure/

37 single-blind studies/

38 placebo/

39 placebos/

40 (random* or sham or placebo*).ti,ab,hw,kw.

41 ((singl* or double*) adj (blind* or dumm* or mask*)).ti,ab,hw,kw.

42 ((tripl* or trebl*) adj (blind* or dumm* or mask*)).ti,ab,hw,kw.

43 random*.ab,ti or placebo*.de,ab,ti or double NEXT1 blind*.ab,ti.

44 or/21-43

45 20 and 44

Medline Search:

1 anticoagula*.mp.

2 exp Anticoagulants/

3 anti?platelet.mp.

4 exp Platelet Aggregation Inhibitors/

5 aspirin.mp.

6 acetylsalicylic acid.mp.

7 exp Stroke/

8 stroke prevention.mp.

9 stroke recurrence.mp.

10 exp Ischemic Attack, Transient/

11 cryptogenic stroke.mp.

12 embolic stroke of undetermined source.mp.

13 embolic stroke of unknown source.mp.

14 ESUS.mp.

15 antithrombin.mp. or exp *Antithrombins/

16 1 or 2 or 15

17 3 or 4 or 5 or 6

18 7 or 8 or 9 or 10

19 11 or 12 or 13 or 14

20 16 and 17 and 18 and 19

21 randomized controlled trial.pt.

22 controlled clinical trial.pt.

23 randomized.ab.

24 placebo.ab.

25 clinical trials as topic.sh.

26 randomly.ab.

27 trial.ti.

28 21 or 22 or 23 or 24 or 25 or 26 or 27

29 exp animals/ not humans.sh.

30 28 not 29

31 20 and 30

Supplemental Tables

<!--Col Count:3-->	Hart et al. (2018)	Diener et al. (2019)
Inclusion Criteria	• Age ≥ 50 years<br>• Patients with age 50-59 years have at least one additional	• Age ≥ 60 years OR: Age 50-59 years with additional risk factors for stroke

<!--Col Count:3-->	Hart et al. (2018)	Diener et al. (2019)
	risk factor for stroke<br>• Non-lacunar ischemic stroke visualized by Computed Tomography (CT) or Magnetic Resonance Image (MRI)<br>• Time from index stroke to randomization and first intake of study medication is between 7 days and 6 months<br>• Absence of cervical carotid atherosclerotic stenosis > 50% or occlusion<br>• No intra-cardiac thrombus on either transesophageal or transthoracic echocardiography<br>• Absence of AF > 6 minutes in duration after ≥ 24-hour cardiac monitoring with automated rhythm detection<br>• All planned diagnostic tests for stroke must be completed	• Non-lacunar Ischemic stroke with brain lesion visualized by Computed Tomography (CT) or Magnetic Resonance Image (MRI)<br>• Index stroke occurred up to 3 months before randomization (mRS ≤3) or up to 6 months before randomization (≤3) in patients ≥ 60 years old with at least one additional risk factor for recurrent stroke<br>• Arterial imaging or cervical plus TCD ultrasonography shows absence of extracranial/intracranial atherosclerosis causing ≥50% luminal stenosis in artery supplying area of recent brain ischemia<br>• Absence of AF > 6 minutes in duration after≥ 24-hour cardiac monitoring with automated rhythm detection
Exclusion Criteria	• Severe disabling stroke (mRS ≥ 4)<br>• Indication for anticoagulation or antiplatelet therapy<br>• Estimated glomerular filtration late (eGFR) < 30 mL/min/1.73 m2	• Severe disabling stroke (mRS ≥ 4) or inability to swallow medications<br>• Indication for anticoagulant therapy<br>• Major risk factors for cardioembolic source of embolism<br>• No other specific stroke etiology<br>• Renal impairment with estimated glomerular filtration late (eGFR) < 30 mL/min/1.73 m2

Table I. Eligibility criteria of the included randomized controlled trials.

<!--Col Count:10-->Study	N	Mean age (years)	Sex (% male)	Anticoagulant group (mg)	Antiplatelet group (mg)	Primary efficacy outcome	Primary safety outcome	Measure of effect	Median follow-up period (months)
Hart et al. (2018)	7213	67	62	Rivaroxaban <!--Soft-enter Run-on-->(15 mg od)	Aspirin (100 mg od)	Recurrent Stroke	Major bleeding	Hazard ratio and 95% confidence interval	11

<!--Col Count:10-->Study	N	Mean age (years)	Sex (% male)	Anticoagulant group (mg)	Antiplatelet group (mg)	Primary efficacy outcome	Primary safety outcome	Measure of effect	Median follow-up period (months)
Diener et al. (2019)	5390	64.2	63	Dabigatran (150 mg or 110 mg bid)	Aspirin (100 mg od)	Recurrent Stroke	Major bleeding	Hazard ratio and 95% confidence interval	19

Table II. Study characteristics of the included randomized controlled trials.

<!--Col Count:5-->Characteristics	Hart et al. (2018)		Deiner et al. (2019)	
	Rivaroxaban	Aspirin	Dabigatran	Aspirin
Number of patients (N)	3609	3604	2695	2695
Race — no. (%)				
White	2612 (72)	2604 (72)	1926 (71.5)	1966 (72.9)
Black	51 (1)	60 (2)	54 (2.0)	40 (1.5)
Asian	716 (20)	698 (19)	631 (23.4)	597 (22.2)
Other	230 (6)	242 (7)	84 (3.1)	92 (3.4)
Median NIHSS score (IQR)	1 (0–2)	1 (0–2)	1 (0–2)	1 (0–2)
Median days from index stroke to randomization (IQR)	38.0 (15.0–89.0)	36.0 (14.0–86.5)	46.0 (21.0–82.0)	43.0 (20.0–78.0)
Median score on modified Rankin Scale (IQR)	1 (0–2)	1 (0–2)	1 (0–2)	1 (0–2)
Mean body mass index (kg/m2)	27.1 ± 4.9	27.3 ± 5.1	27.2 ± 5.0	27.3 ± 5.0
Diabetes mellitus — no. (%)	889 (25)	917 (25)	585 (21.7)	639 (23.7)
Previous stroke or TIA — no. (%)	620 (17)	643 (18)	475 (17.6)	500 (18.6)
Hypertension — no. (%)	2782 (77)	2803 (78)	1996 (74.1)	1985 (73.7)
Current tobacco use - no. (%)	756 (21)	728 (20)	458 (17.0)	433 (16.1)

Table III. Patient characteristics of the included randomized controlled trials.

<!--Col Count:6-->Study	Sequence generation	Allocation concealment	Blinding of participants, personal and outcome assessors	Incomplete outcome data	Selective outcome reporting
Hart et al.	Low	Low	Low	Low	Low
Diener et al.	Low	Low	Low	Low	Low

Table IV. Risk of bias assessment of the included randomized controlled trials.

<!--Col Count:9-->Outcomes	Hart et al. (2018)				Diener et al. (2019)			
	Anticoagulant - no. (%)	Antiplatelet - no. (%)	Hazard ratio	CI	Anticoagulant - no. (%)	Antiplatelet - no. (%)	Hazard ratio	CI
Recurrent stroke	171 (5.1)	158 (4.7)	1.08	0.87–1.34	177 (4.1)	207 (4.8)	0.85	0.69–1.03
Ischemic stroke	158 (4.7)	156 (4.7)	1.01	0.81–1.26	172 (4.0)	203 (4.7)	0.84	0.68–1.03
Disabling stroke	41 (1.2)	29 (0.8)	1.42	0.88–2.28	25 (0.6)	42 (0.9)	0.59	0.36–0.96
All-cause mortality	65 (1.9)	52 (1.5)	1.26	0.87–1.81	56 (1.2)	58 (1.3)	0.96	0.66–1.38
Hemorrhagic stroke	13 (0.4)	2 (0.1)	6.5	1.47–28.8	6 (0.1)	7 (0.2)	0.86	0.29–2.55
Systemic embolism	1 (<0.1)	2 (0.1)	0.5	0.05–5.51	6 (0.1)	11 (0.2)	0.54	0.20–1.46
Myocardial infarction	17 (0.5)	23 (0.7)	0.74	0.39–1.38	23 (0.5)	18 (0.4)	1.28	0.69–2.38
Major bleeding	62 (1.8)	23 (0.7)	2.72	1.68–4.39	62 (1.8)	23 (0.7)	2.72	1.68–4.39
Clinically relevant nonmajor bleeding	118 (3.5)	79 (2.3)	1.51	1.13–2.00	118 (3.5)	79 (2.3)	1.51	1.13–2.00

Table V. The incidence of efficacy and safety outcomes from the included randomized controlled trials.

Conflicting Interests: In the last 3 years HCD received honoraria for participation in clinical trials, contribution to advisory boards or oral presentations from: Abbott, BMS, Boehringer Ingelheim, Daiichi-Sankyo, Novo-Nordisk, Pfizer, Portola and WebMD Global. Financial support for research projects was provided by Boehringer Ingelheim. HCD received research grants from the German Research Council (DFG), German Ministry of Education and Research (BMBF), European Union, NIH, Bertelsmann Foundation and Heinz-Nixdorf Foundation. JWE has received honoraria and/or research support from Bayer, Boehringer Ingelheim, Bristol Myers Squibb, Daiichi-Sankyo, Janssen, Pfizer, Portola and WebMD Global.

Sources of Funding: This systematic review and meta-analysis did not receive any funding.

Informed Consent: Not applicable, Ethical Approval: Not applicable, Guarantor: JWE

Contributorship: RGD, KSP and JWE conceived the study. NNH, KP, OS, KSP, RGD and JWE were involved in protocol development. NNH, KP, and OS search the literature, extracted and analyzed the data and wrote the first draft of the manuscript. All authors reviewed and edited the manuscript and approved the final version of the manuscript.
